# Next generation sequencing of HIV-1 protease in the PIVOT trial of protease inhibitor monotherapy

**DOI:** 10.1016/j.jcv.2018.02.003

**Published:** 2018-04

**Authors:** David T. Dunn, Wolfgang Stöhr, Alejandro Arenas-Pinto, Anna Tostevin, Jean L. Mbisa, Nicholas I. Paton, or the Protease Inhibitor Monotherapy Versus Ongoing Triple Therapy PIVOT Trial Team

**Affiliations:** a MRC Clinical Trials Unit at UCL, London, UK; b Institute for Global Health, UCL, London, UK; c Public Health England, London, UK; d Yong Loo Lin School of Medicine, National University of Singapore, Singapore

**Keywords:** HIV, Protease inhibitor, Resistance, NGS

## Abstract

**Background:**

The PIVOT trial examined whether patients with suppressed viral load on combination antiretroviral therapy could be safely switched long-term to ritonavir-boosted protease inhibitor (PI) monotherapy. The main trial publication reported that only one of 296 patients allocated to PI monotherapy experienced a loss of drug options due to protease mutations (identified by local Sanger sequencing resistance tests) likely selected by study drug.

**Objectives:**

To assess if we had missed low frequency mutations, using a more sensitive methodology.

**Study design:**

We performed next generation sequencing (NGS) on all available frozen plasma samples with VL >1000 copies/ml from patients who were randomised to PI monotherapy. Assays were performed at Public Health England laboratories using a previously described method.

Median coverage depth was 76,000 and the threshold for detection of minority variants was 2%. Drug susceptibility was predicted using the Stanford HIVdb algorithm.

**Results:**

17 of 26 potential samples, all from different patients, were identified and successfully tested. The median viral load was 6780 copies/ml and the median time since randomisation was 43 weeks. NGS revealed previously unidentified minority variant protease mutations (G73D, I54T, L89V) in three samples, at frequencies ranging between 2% and 10%. None of these mutations predicted intermediate or high level resistance, the trial primary outcome.

**Discussion:**

This report adds to the body of evidence that ritonavir-boosted PI monotherapy, when used as a switch strategy with prompt detection of viral load rebound and early re-introduction of combination therapy, rarely leads to the development of clinically important protease resistance mutations.

## Highlights

• Next generation sequencing (NGS) was performed on patients on PI monotherapy.
• It revealed previously unidentified minority variant protease mutations in 3 samples.
• These mutations do not predict clinically important phenotypic resistance.

## 1 Background

The UK-based PIVOT trial examined whether patients with suppressed viral load (VL) on combination antiretroviral therapy could be safely switched to ritonavir-boosted protease inhibitor (PI) monotherapy, with regular HIV VL monitoring and prompt reintroduction of combination therapy for VL rebound (defined as a confirmed VL ≥50 copies/ml) [1]. The primary outcome in the trial was the loss of future drug options, defined as intermediate to high level resistance (predicted from genotype) to one or more licensed drugs in contemporary use. Resistance tests were performed at site laboratories using various Sanger sequencing assays.

Of the 296 patients who were allocated to PI monotherapy, 105 were genotyped at least once (153 resistance tests in total), and the primary outcome observed in six patients. Three of these six patients had reverse transcriptase mutations but no protease mutations. In two other patients, both of whom received darunavir monotherapy and had no prior PI exposure, L90M was the sole protease mutation detected; this is rarely selected de novo by darunavir and is a relatively common transmitted mutation [[2], [3]]. It appears therefore that only a single PI monotherapy patient (described below, patient A) experienced a loss of drug options due to a mutation (I50L) selected by study drug. However, a limitation of Sanger sequencing is its inability to detect viral variants below a threshold of approximately 20%.

### 1.1 Objectives

To examine whether the main analysis of PIVOT had missed low frequency mutations, by applying a more sensitive sequencing methodology to selected stored samples.

### 1.2 Study design

We performed next generation sequencing (NGS) on all available frozen plasma samples with VL >1000 copies/ml (irrespective of whether the formal definition of virological failure was fulfilled) that had been stored from patients who were randomised to PI monotherapy. NGS detects the same range of mutants than Sanger sequencing but with much higher sensitivity. Assays were performed at Public Health England, with sequencing performed using MiSeq Reagent Kit version 2 (Illumina) [4]. The threshold for low-frequency variant detection was set at 2%.

## 2 Results

Of 26 potential samples, 17 (all from different patients) were identified and successfully tested. One patient received atazavair, two patients lopinavir, and 14 patients darunavir, as ritonavir-boosted monotherapy. The median (IQR) HIV VL was 6780 (3333–10,729) copies/ml, the median (IQR) time since randomisation was 43 (33–53) weeks, and the median (IQR) interval between first detectable VL and the date of sample was 6 (4–8) weeks. 10 of the samples had previously been tested by Sanger sequencing from the same blood draw; a paired comparison showed 99.2% homology at the nucleotide level, using a NGS variant detection level of 20%. The 7 remaining samples were from patients who had been tested by Sanger sequencing at a different time point. Protease mutations were defined as major or accessory, and drug susceptibility predicted using the Stanford HIVdb algorithm (version 8.1.1) (https://hivdb.stanford.edu/).

NGS (median coverage depth 76,000, range 20,000–170,000) revealed minority variant mutations in three of the 17 patients tested, all of whom were infected with subtype C virus (Table 1). Patient A, who received atazanavir as PI monotherapy, met the primary outcome due to an I50L mutation (patient 5 in Table 2 in main trial report [1]). NGS showed a previously undetected G73D mutation (frequency 10%), predicted to confer resistance to saquinavir (i.e. Stanford penalty score ≥10), at week 57. Notably, I50L was not detected as a minority variant at this time point despite its detection (as a mixture) by Sanger sequencing at week 48, and it was also absent by Sanger sequencing at week 71. This suggests that the mutation may have been stochastic rather than a result of drug selection pressure. The patient remained on atazanavir monotherapy throughout the period spanned by these three tests. The virus of patient B, who received darunavir monotherapy, expressed the I54T mutation (frequency 2%) at 48 weeks. Paradoxically, this mutation predicts resistance to all PIs other than darunavir, again calling into question whether it actually arose from drug selection pressure (further supported by the low level of this variant). The L89V mutation (frequency 5%), which predicts resistance to fosamprenavir, was detected at 41 weeks in patient C. This patient simplified treatment from lopinavir/tenofovir/emtricitabine to lopinavir monotherapy at trial entry. Given the inability to test patients at baseline, it is not possible to conclude whether the mutation emerged before or after trial entry.

**Table 1:** Resistance test results on the three patients with protease mutations additionally identified by NGS.

Subject	Drugs received before PIVOT	Regimen during PIVOT^a^	Weeks since randomisation	Viral load (copies/ml)	Protease mutations by Sanger sequencing^b^	Protease mutations by NGS^b^	Predicted PI resistance^c^
A	TDF, FTC, EFV	ATV/r	48	23,400	I50I/L, K20K/T	ND	ATV(H)
			57	3300	K20K/T	K20T (13%; 99,917), G73D (10%; 76,126)	ATV(PL),FPV(PL),SQV(L)^d^
			61	2400	None	ND	
B	ZDV, TDF, 3TC, FTC, EFV	DRV/r	48	35,431	ND	I54T (2%; 112,532)	ATV (L), LPV(L)
			52	17,700	None	ND	
C	TDF, FTC, LPV/r	LPV/r	41	10,700	None	L89V (5%; 98,051)	FPV(PL)

ND = not done.

a All three subjects remained on same regimens from randomisation to date of resistance test samples.

b Major or accessory mutations according to Stanford HIVdb Version 8.1.1. I50L is major mutation; all other listed mutations are accessory. Values in parentheses are frequency of mutation; depth of read at that position.

c Predicted by Stanford HIVdb Version 8.1.1, based on consensus NGS sequence (preferentially) or Sanger sequence. PL = potential low level, L = low level, H = high.

d Predicted to be susceptible to all drugs by mutations detected by Sanger sequencing.

In summary, NGS did not identify any additional patients who met the trial primary outcome of intermediate or high level resistance. Furthermore, the phenotypic impact of low-level variants may be over-estimated by the Stanford algorithm, which was developed in the context of majority variants.

## 3 Discussion

This report adds to the body of evidence that ritonavir-boosted PI monotherapy rarely leads to the emergence of protease resistance mutations when used as a switch strategy with prompt detection of VL rebound and early re-introduction of effective combination therapy. The risk may be higher in a routine clinical study setting, where monitoring may be less intensive. However, studies of second-line PI monotherapy in settings without VL monitoring have found that protease resistances emerges slowly [[5], [6]] Another sensitive genotyping method, single genome sequencing (SGS), was used in two other trials of maintenance PI monotherapy. In the MONOI study, 9 patients who received darunavir PI monotherapy were tested; additional protease resistance mutations (at positions 32, 47, 50) were found on 1/50 viral clones from one patient [7]. In the OK04 study, SGS was performed on 11 patients who received lopinavir monotherapy; additional mutations were detected on 3/45 viral clones at positions 46 and 82 in two patients [8].

In spite of the substantial evidence of safety, PI monotherapy has failed to achieve wide clinical acceptance as a maintenance strategy in patients with established viral suppression, possibly due to a higher overall risk of virological rebound compared with combination therapy. However, recent analyses have shown that patients at lower risk of virological rebound can be readily identified [9] and that intensification of therapy achieves rapid re-suppression without development of resistance [[1], [5]]. It is also important to note that PI monotherapy precludes the development of resistance to NRTIs and NNRTIs. As drugs from these classes generally have a lower barrier to resistance, the overall burden of resistance in the long run may be less for PI monotherapy than for combination therapy. Finally, recent simplification trials have shown that dual therapy regimens consisting of a PI plus lamivudine are as effective as ongoing triple therapy in terms of short-term viral suppression [[10], [11]]. Our finding of minimal protease resistance with PI monotherapy should also generalise to these more potent regimens.

## Funding

This work was supported by the National Institute for Health Research Health Technology Assessment programme (project number 06/403/90) and the Medical Research Council (grant MC_UU_12023/23). The views and opinions expressed herein are those of the authors and do not necessarily reflect those of the HTA programme, NIHR, NHS or the Department of Health.

## Competing interests

NP, WS, DTD, and AA-P report grants from the National Institute of Health Research Health Technology Assessment programme during the conduct of this study. NP reports grants, personal fees, and non-financial support from AbbVie and Janssen; personal fees and non-financial support from Merck and Roche; grants and non-financial support from GlaxoSmithKline; and non-financial support from Gilead Sciences, all outside the submitted work. AA-P reports grants from Janssen and ViiV Healthcare outside the submitted work.

## Ethical approval

The trial protocol was approved by the Cambridgeshire 4 Research Ethics Committee and Medicines and Healthcare Products Regulatory Agency.
